# Supplementary material for: The Small RNA Universe of Capitella teleta
Source: Front Mol Biosci. 2022 Feb 25;9:802814. doi: 10.3389/fmolb.2022.802814 (PMC8915122; doi:10.3389/fmolb.2022.802814)
Supplement: Supplementary file 1 [file DataSheet1.ZIP › Supplement/known/CAPTEscaffold_24_2928.pdf]

| | | |
|---|---|---|
| Provisional ID | : | CAPTEscaffold_24_2928 |
| Score total | : | 32121 |
| Score for star read(s) | : | 3.9 |
| Score for read counts | : | 32114 |
| Score for mfe | : | 2.1 |
| Score for randfold | : | 1.6 |
| Score for cons. seed | : | -0.6 |
| Total read count | : | 63002 |
| Mature read count | : | 43788 |
| Loop read count | : | 4 |
| Star read count | : | 19210 |

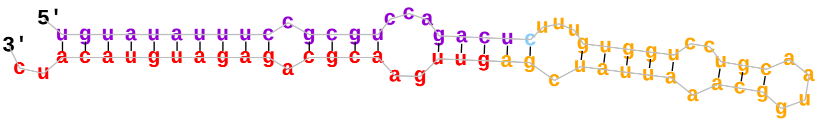


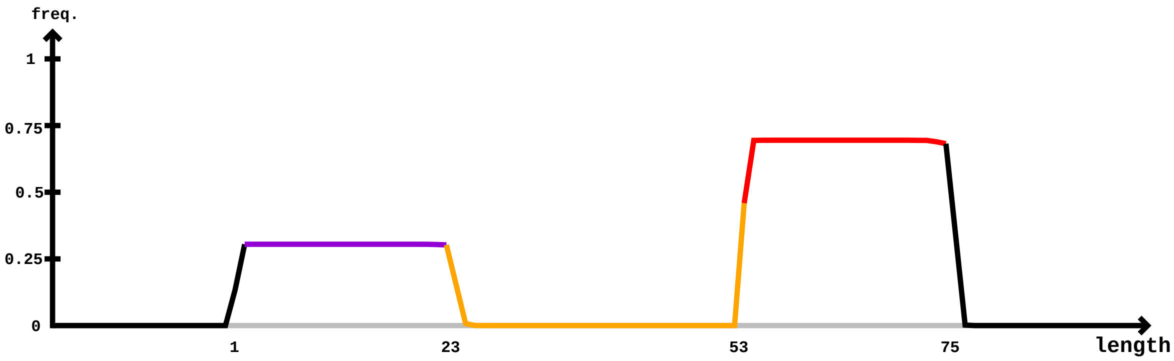


Star Mature

```
5'- aucugcugggucugguggguguauauuuccgcguccagacucuuugugguccugcaauggcaaauuaucgaguugaacgcagagauguacaucaccaaccgcagaccaaauca  -3'   obs
    aucugcugggucugguggguguauauuuccgcguccagacucuuugugguccugcaauggcaaauuaucgaguugaacgcagagauguacaucaccaaccgcagaccaaauca         exp
    aucugcugggucugguggguguauauuuccgcguccagacucuuugugguccugcaauggcaaauuaucgaguugaacgcagagauguacaucaccaaccgcagaccaaauca         known
    .(((((..(((.((((((((((((((((.((((...(((((...(((((..(((....))).))))).)))))..)))).))))))))))))))))))))))))........  reads  mm  sample
    ...............Cguguauauuuccgcguccagacu.........................................................................  1      1   seq
    ................guguauauuuccgcgucc..............................................................................  1      0   seq
    ................guguauauuuccgcguccag............................................................................  1      0   seq
    ................guguauauuuccgcguccaga...........................................................................  32     0   seq
    ................guguauauuuccAcguccaga...........................................................................  1      1   seq
    ................guguauauuuccgcguccagac..........................................................................  35     0   seq
    ................guguauaAuuccgcguccagacu.........................................................................  2      1   seq
    ................guguauauuuAcgcguccagacu.........................................................................  2      1   seq
    ................guguauauuuccgcgAccagacu.........................................................................  1      1   seq
    ................guguauauuuccgcguUcagacu.........................................................................  1      1   seq
    ................guguauauuuccgcAuccagacu.........................................................................  3      1   seq
    ................guguauauuuUcgcguccagacu.........................................................................  83     1   seq
    ................guguauauuuccgcgucAagacu.........................................................................  2      1   seq
    ................guguauauuuccgAguccagacu.........................................................................  2      1   seq
    ................guguauauuuccgcguccagacu.........................................................................  8140   0   seq
    ................guguauauuuccgcgucUagacu.........................................................................  7      1   seq
    ................guUuauauuuccgcguccagacu.........................................................................  1      1   seq
    ................gugCauauuuccgcguccagacu.........................................................................  1      1   seq
    ................gugAauauuuccgcguccagacu.........................................................................  2      1   seq
    ................guguauauAuccgcguccagacu.........................................................................  2      1   seq
    ................guguauGuuuccgcguccagacu.........................................................................  1      1   seq
    ................guguaAauuuccgcguccagacu.........................................................................  3      1   seq
    ................guAuauauuuccgcguccagacu.........................................................................  2      1   seq
    ................Nuguauauuuccgcguccagacu.........................................................................  6      1   seq
    ................guguauauuuccgcguccagacA.........................................................................  11     1   seq
    ................guguauauuuccgcguccaAacu.........................................................................  1      1   seq
    ................Auguauauuuccgcguccagacu.........................................................................  2      1   seq
    ................guguauauuuccgcguccagaAu.........................................................................  1      1   seq
    ................guguauauuuccgcguccagacC.........................................................................  1      1   seq
    ................guguauauuuccgcguccagaUu.........................................................................  2      1   seq
    ................gGguauauuuccgcguccagacu.........................................................................  1      1   seq
    ................guguauauuuccgcguccagacG.........................................................................  14     1   seq
```

Star Mature

| | | | |
|---|---|---|---|
| aucugcugggucugguggguauauuuccgcguccagacucuuugugguccugcaauggcaaauuaucgaguugaacgcagagauguacaucaccaaccgcagaccaaauca | | | |
| .................gAguauauuuccgcguccagacu........................................................................ | 3 | 1 | seq |
| .................guguauauuuccgcguccUgacu........................................................................ | 1 | 1 | seq |
| .................guguauauuuccAcguccagacu........................................................................ | 1 | 1 | seq |
| .................guguGuauuuccgcguccagacu........................................................................ | 1 | 1 | seq |
| .................guguauauuucUgcguccagacu........................................................................ | 3 | 1 | seq |
| .................guguauauuucAgcguccagacu........................................................................ | 2 | 1 | seq |
| .................guguauauuuccgcguccaUacu........................................................................ | 1 | 1 | seq |
| .................Uuguauauuuccgcguccagacu........................................................................ | 19 | 1 | seq |
| .................guguauauuuccgUguccagacu........................................................................ | 1 | 1 | seq |
| .................guguauauuAccgcguccagacu........................................................................ | 1 | 1 | seq |
| .................guguaCauuuccgcguccagacu........................................................................ | 2 | 1 | seq |
| .................guguauauuuccgcgCccagacu........................................................................ | 1 | 1 | seq |
| .................guguauauuuccgcguccagacuc....................................................................... | 3 | 0 | seq |
| .................guguauauuuccgcguccagacuA....................................................................... | 1 | 1 | seq |
| .................guguauauuuccgcguccagacuU....................................................................... | 3 | 1 | seq |
| .................guguauauuuccgcguccagacuUu...................................................................... | 1 | 1 | seq |
| .................guguauauuuccgcguccagacucu...................................................................... | 4 | 0 | seq |
| .................guguauauuuccgcguccagacuUuu..................................................................... | 4 | 1 | seq |
| .................guguauauuuccgcguccagacucuug.................................................................... | 1 | 0 | seq |
| ..................uguauauuuccgcguccag........................................................................... | 8 | 0 | seq |
| ..................uguauauuuccgcguccaga.......................................................................... | 23 | 0 | seq |
| ..................uguauauuuccgcguccagac......................................................................... | 50 | 0 | seq |
| ..................uAuauauuuccgcguccagacu........................................................................ | 2 | 1 | seq |
| ..................uguauauuAccgcguccagacu........................................................................ | 2 | 1 | seq |
| ..................uguauauuuccgcAuccagacu........................................................................ | 7 | 1 | seq |
| ..................ugGauauuuccgcguccagacu........................................................................ | 1 | 1 | seq |
| ..................uguauauuuccUcguccagacu........................................................................ | 2 | 1 | seq |
| ..................Aguauauuuccgcguccagacu........................................................................ | 22 | 1 | seq |
| ..................Gguauauuuccgcguccagacu........................................................................ | 7 | 1 | seq |
| ..................uguGuauuuccgcguccagacu........................................................................ | 2 | 1 | seq |
| ..................uguauauuuccgcguAcagacu........................................................................ | 2 | 1 | seq |
| ..................uUuauauuuccgcguccagacu........................................................................ | 1 | 1 | seq |
| ..................uguauUuuuccgcguccagacu........................................................................ | 2 | 1 | seq |
| ..................uguauauuuccgcgucUagacu........................................................................ | 10 | 1 | seq |
| ..................uguauauuuUcgcguccagacu........................................................................ | 1 | 1 | seq |
| ..................Cguauauuuccgcguccagacu........................................................................ | 1 | 1 | seq |
| ..................uguauauuuccCcguccagacu........................................................................ | 1 | 1 | seq |
| ..................uguauauuuGcgcguccagacu........................................................................ | 2 | 1 | seq |
| ..................uguauauuuccgcguccagacG........................................................................ | 18 | 1 | seq |
| ..................uguauauuucAgcguccagacu........................................................................ | 2 | 1 | seq |
| ..................uguauauuuccgcguccagacC........................................................................ | 1 | 1 | seq |
| ..................uguauaAuuccgcguccagacu........................................................................ | 1 | 1 | seq |
| ..................uguauauuuccgUguccagacu........................................................................ | 3 | 1 | seq |
| ..................uguauauAuccgcguccagacu........................................................................ | 3 | 1 | seq |
| ..................uguauauuuccgcguccaUacu........................................................................ | 2 | 1 | seq |
| ..................ugAauauuuccgcguccagacu........................................................................ | 7 | 1 | seq |
| ..................uguauauuuccgcguccagacu........................................................................ | 8872 | 0 | seq |
| ..................uguauauuuccgcguccagacA........................................................................ | 14 | 1 | seq |
| ..................uguaAauuuccgcguccagacu........................................................................ | 3 | 1 | seq |
| ..................uguauGuuuccgcguccagacu........................................................................ | 1 | 1 | seq |
| ..................Nguauauuuccgcguccagacu........................................................................ | 3 | 1 | seq |
| ..................uguauaGuuccgcguccagacu........................................................................ | 1 | 1 | seq |
| ..................uguauauuuccgcgCccagacu........................................................................ | 1 | 1 | seq |
| ..................uguauauuucGgcguccagacu........................................................................ | 2 | 1 | seq |
| ..................uguauauuuAcgcguccagacu........................................................................ | 6 | 1 | seq |
| ..................uguauauuuccgGguccagacu........................................................................ | 1 | 1 | seq |
| ..................uguauauuucUgcguccagacu........................................................................ | 7 | 1 | seq |
| ..................uguauauuuccgcguccGgacu........................................................................ | 4 | 1 | seq |
| ..................uguauauuuccgcgucAagacu........................................................................ | 1 | 1 | seq |
| ..................uguUuauuuccgcguccagacu........................................................................ | 1 | 1 | seq |
| ..................uguauauuuccgcguUcagacu........................................................................ | 1 | 1 | seq |
| ..................uguauauuuccgcguccagUcu........................................................................ | 2 | 1 | seq |
| ..................ugAauauuuccgcguccagacuc....................................................................... | 3 | 1 | seq |
| ..................uguauauuAccgcguccagacuc....................................................................... | 1 | 1 | seq |
| ..................uguauauuuccgcguccagacuc....................................................................... | 1210 | 0 | seq |
| ..................uguauauuuccgcgucUagacuc....................................................................... | 1 | 1 | seq |
| ..................uguauauuuccgcguccagaGuc....................................................................... | 1 | 1 | seq |
| ..................uguauauuucAgcguccagacuc....................................................................... | 1 | 1 | seq |
| ..................uguauauuuccAcguccagacuc....................................................................... | 1 | 1 | seq |
| ..................uguauauuuccgUguccagacuc....................................................................... | 1 | 1 | seq |

Star Mature

```
aucugcugggucugguggguauauuuccgcguccagacucuuugugguccugcaauggcaaauuaucgaguugaacgcagagauguacaucaccaaccgcagaccaaauca
..................uguauauuuAcgcguccagacuc.......................................................................    1    1    seq
..................uguauauuucUgcguccagacuc.......................................................................    2    1    seq
..................uguauaAuuccgcguccagacuc.......................................................................    1    1    seq
..................Aguauauuuccgcguccagacuc.......................................................................    3    1    seq
..................ugCauauuuccgcguccagacuc.......................................................................    1    1    seq
..................uguauauuuccgcguccagGcuc.......................................................................    1    1    seq
..................uguaAauuuccgcguccagacuc.......................................................................    1    1    seq
..................uguauauuuccgcguccagacuU.......................................................................    1    1    seq
..................uguauauCuccgcguccagacuc.......................................................................    1    1    seq
..................uguauauuuccgcgCccagacuc.......................................................................    1    1    seq
..................Gguauauuuccgcguccagacuc.......................................................................    2    1    seq
..................uguauauAuccgcguccagacuc.......................................................................    3    1    seq
..................uguaCauuuccgcguccagacucu......................................................................    3    1    seq
..................uguauauuuccgcguccagacuAu......................................................................    1    1    seq
..................uguauauuuccgcguccagacucA......................................................................    28   1    seq
..................uguaAauuuccgcguccagacucu......................................................................    2    1    seq
..................uguauGuuuccgcguccagacucu......................................................................    1    1    seq
..................uguauauuuccgcguccagacucG......................................................................    3    1    seq
..................uguauauuuccgcguccagacuUu......................................................................    1    1    seq
..................uguauauuuAcgcguccagacucu......................................................................    1    1    seq
..................uguauauuuccgcguccagacucu......................................................................    403  0    seq
..................uguauauuuccgcguccagacucuA.....................................................................    1    1    seq
..................uguauauuuccgcguccagacucuu.....................................................................    1    0    seq
..................uguauauuuccgcguccagacucuuu....................................................................    1    0    seq
..................uguauauuuccgcguccagacucuuugu..................................................................    1    0    seq
...................guauauuuccgcguccagacu........................................................................    2    0    seq
....................uauauuuccgcguccagacuc.......................................................................    2    0    seq
....................uauauuuccgcguccagacucu......................................................................    1    0    seq
........................................cuuugugguccugcaauggcaaauuaucgag.........................................    3    0    seq
.........................................uuugugguccugcaauggcaaauuauU............................................    1    1    seq
......................................................................guugaacgcagagaugua........................    34   0    seq
......................................................................guugaacgcGgagaugua........................    2    1    seq
......................................................................guugaacgcGgagauguac.......................    1    1    seq
......................................................................guugaacgcagagauguac.......................    7    0    seq
......................................................................guCgaacgcagagauguaca......................    1    1    seq
......................................................................guugaacgUagagauguaca......................    11   1    seq
......................................................................guAgaacgcagagauguaca......................    1    1    seq
......................................................................Uuugaacgcagagauguaca......................    1    1    seq
......................................................................guuAaacgcagagauguaca......................    1    1    seq
......................................................................guuUaacgcagagauguaca......................    1    1    seq
......................................................................guugaacgcagagauguGca......................    1    1    seq
......................................................................guugaacgcGgagauguaca......................    29   1    seq
......................................................................guugaacgcagagauguaca......................    240  0    seq
......................................................................guugaacgcaAagauguacau.....................    1    1    seq
......................................................................Uuugaacgcagagauguacau.....................    2    1    seq
......................................................................guAgaacgcagagauguacau.....................    1    1    seq
......................................................................guugaacgcagagauguacau.....................    380  0    seq
......................................................................guugaacgcGgagauguacau.....................    21   1    seq
......................................................................guugaacgUagagauguacau.....................    11   1    seq
......................................................................guugaacgcagaCauguacau.....................    1    1    seq
......................................................................guugaaAgcagagauguacau.....................    1    1    seq
......................................................................guugaacgcagagauCuacau.....................    1    1    seq
......................................................................guugaacgcagagauguacGuc....................    5    1    seq
......................................................................guugaacgcagagauguUcauc....................    2    1    seq
......................................................................guugaacgcCgagauguacauc....................    1    1    seq
......................................................................guugaacgcagagaCguacauc....................    4    1    seq
......................................................................guugaacgcagagauguacauA....................    12   1    seq
......................................................................Auugaacgcagagauguacauc....................    4    1    seq
......................................................................guugaacgcagagauUuacauc....................    4    1    seq
......................................................................gCugaacgcagagauguacauc....................    4    1    seq
......................................................................guuAaacgcagagauguacauc....................    46   1    seq
......................................................................guugaacCcagagauguacauc....................    3    1    seq
......................................................................guugaGcgcagagauguacauc....................    15   1    seq
......................................................................Cuugaacgcagagauguacauc....................    6    1    seq
......................................................................gAugaacgcagagauguacauc....................    14   1    seq
......................................................................guuCaacgcagagauguacauc....................    5    1    seq
......................................................................guugaacgcagaAauguacauc....................    3    1    seq
......................................................................guugaacgcagagauguaUauc....................    5    1    seq
......................................................................guugaacgcaAagauguacauc....................    13   1    seq
......................................................................guugaacgcagaUauguacauc....................    5    1    seq
```

**Star** **Mature**

```
aucugcugggucuggugguguauauuuccgcguccagacucuuugugguccugcaauggcaaauuaucgaguugaacgcagagauguacaucaccaaccgcagaccaaauca
......................................................................guNgaacgcagagauguacauc....................    1       1    seq
......................................................................guugaacgcagagauguacaCc....................    1       1    seq
......................................................................guugaacgcagagauguacaAc....................    9       1    seq
......................................................................guugaacgcagagauguGcauc....................    4       1    seq
......................................................................gGugaacgcagagauguacauc....................    9       1    seq
......................................................................guugaacgcagaCauguacauc....................    2       1    seq
......................................................................guugaacgcGgagauguacauc....................    2416    1    seq
......................................................................guugaacgcaUagauguacauc....................    1       1    seq
......................................................................guugaacgcagagUuguacauc....................    2       1    seq
......................................................................guugaacgcagagaugCacauc....................    3       1    seq
......................................................................guAgaacgcagagauguacauc....................    33      1    seq
......................................................................guugaacgcagagaGguacauc....................    1       1    seq
......................................................................guugaacgcUgagauguacauc....................    8       1    seq
......................................................................guugaaGgcagagauguacauc....................    3       1    seq
......................................................................guugaacgcagagauCuacauc....................    1       1    seq
......................................................................guugaacgcagagauguacauU....................    19      1    seq
......................................................................guugaacAcagagauguacauc....................    2       1    seq
......................................................................guCgaacgcagagauguacauc....................    9       1    seq
......................................................................guugUacgcagagauguacauc....................    3       1    seq
......................................................................Uuugaacgcagagauguacauc....................    43      1    seq
......................................................................guugaacgcagagauAuacauc....................    4       1    seq
......................................................................guugaacgcagagauguCcauc....................    1       1    seq
......................................................................guugaacgcagagauguacUuc....................    3       1    seq
......................................................................guugaaAgcagagauguacauc....................    8       1    seq
......................................................................guugaacgcagagauguacauc....................    25212   0    seq
......................................................................guugaacgcagagauguaAauc....................    1       1    seq
......................................................................guugaCcgcagagauguacauc....................    1       1    seq
......................................................................guugaacgAagagauguacauc....................    9       1    seq
......................................................................guugaacUcagagauguacauc....................    3       1    seq
......................................................................guugaacgcagagaugAacauc....................    5       1    seq
......................................................................Nuugaacgcagagauguacauc....................    2       1    seq
......................................................................guugaacgUagagauguacauc....................    118     1    seq
......................................................................guugaacgcagagauguacaGc....................    1       1    seq
......................................................................gNugaacgcagagauguacauc....................    1       1    seq
......................................................................guugaUcgcagagauguacauc....................    5       1    seq
......................................................................guugGacgcagagauguacauc....................    6       1    seq
......................................................................guugaacgcagGgauguacauc....................    6       1    seq
......................................................................guuUaacgcagagauguacauc....................    5       1    seq
......................................................................guugaacgcagagaAguacauc....................    5       1    seq
......................................................................Cuugaacgcagagauguacauca...................    2       1    seq
......................................................................guugaacgcagagauguacauca...................    23      0    seq
......................................................................guugaacgcagagauguacaucU...................    21      1    seq
.......................................................................uugaacgcGgagauguaca......................    2       1    seq
.......................................................................uugaacgcagagauguaca......................    31      0    seq
.......................................................................uugaacgcagagauguacau.....................    38      0    seq
.......................................................................uugaacgcagagGuguacauc....................    1       1    seq
.......................................................................uCgaacgcagagauguacauc....................    1       1    seq
.......................................................................uugaacgcagagauguacGuc....................    1       1    seq
.......................................................................uugaaGgcagagauguacauc....................    1       1    seq
.......................................................................uugaacUcagagauguacauc....................    1       1    seq
.......................................................................uugaacgcagagauguacauU....................    1       1    seq
.......................................................................uugaacgcGgagauguacauc....................    342     1    seq
.......................................................................uugaacgcUgagauguacauc....................    2       1    seq
.......................................................................Augaacgcagagauguacauc....................    7       1    seq
.......................................................................uugaacgcagagaugCacauc....................    1       1    seq
.......................................................................uugaaUgcagagauguacauc....................    2       1    seq
.......................................................................uugaacgcagagaugAacauc....................    3       1    seq
.......................................................................uugaacgcagagauguUcauc....................    1       1    seq
.......................................................................uugUacgcagagauguacauc....................    1       1    seq
.......................................................................Nugaacgcagagauguacauc....................    1       1    seq
.......................................................................uugaacgcaUagauguacauc....................    1       1    seq
.......................................................................uuAaacgcagagauguacauc....................    5       1    seq
.......................................................................uugaacgcaCagauguacauc....................    1       1    seq
.......................................................................uAgaacgcagagauguacauc....................    4       1    seq
.......................................................................uugaacgcagaUauguacauc....................    1       1    seq
.......................................................................uugaacgcagagauguacaAc....................    1       1    seq
.......................................................................uugaacgcagagauguacauG....................    1       1    seq
.......................................................................uugaacgcagagauAuacauc....................    1       1    seq
.......................................................................uugaacgcagagauguacaGc....................    2       1    seq
.......................................................................uuCaacgcagagauguacauc....................    1       1    seq
```

## Star

## Mature

| | | | |
|---|---|---|---|
| aucugcugggucuggugguguauauuuccgcguccagacucuuugugguccugcaauggcaaauuaucgaguugaacgcagagauguacaucaccaaccgcagaccaaauca | | | |
| .......................................................................Gugaacgcagagauguacauc.................... | 2 | 1 | seq |
| .......................................................................uugaacgcaAagauguacauc.................... | 2 | 1 | seq |
| .......................................................................uugaacgcagagauCuacauc.................... | 1 | 1 | seq |
| .......................................................................uugaacgcagUgauguacauc.................... | 1 | 1 | seq |
| .......................................................................Cugaacgcagagauguacauc.................... | 1 | 1 | seq |
| .......................................................................uugaaAgcagagauguacauc.................... | 1 | 1 | seq |
| .......................................................................uugaacgcagagauguacauc.................... | 3511 | 0 | seq |
| .......................................................................uugaacgcagagauguacauUa................... | 7 | 1 | seq |
| .......................................................................uugaacUcagagauguacauca................... | 7 | 1 | seq |
| .......................................................................uugaacgcagagauguacaucU................... | 46 | 1 | seq |
| .......................................................................uugaacgcagagauguUcauca................... | 1 | 1 | seq |
| .......................................................................uugaacgcagaAauguacauca................... | 1 | 1 | seq |
| .......................................................................uugaacgcGgagauguacauca................... | 147 | 1 | seq |
| .......................................................................uugaacgcaUagauguacauca................... | 2 | 1 | seq |
| .......................................................................uugaacgcagagaAguacauca................... | 6 | 1 | seq |
| .......................................................................uugaacgcUgagauguacauca................... | 1 | 1 | seq |
| .......................................................................uugaaAgcagagauguacauca................... | 4 | 1 | seq |
| .......................................................................Gugaacgcagagauguacauca................... | 3 | 1 | seq |
| .......................................................................uugaacgcaCagauguacauca................... | 2 | 1 | seq |
| .......................................................................uugaacgcagaUauguacauca................... | 2 | 1 | seq |
| .......................................................................uCgaacgcagagauguacauca................... | 3 | 1 | seq |
| .......................................................................uugaacgcagagauguacaCca................... | 1 | 1 | seq |
| .......................................................................uugaacgcaAagauguacauca................... | 8 | 1 | seq |
| .......................................................................uugaacgcagagaugCacauca................... | 2 | 1 | seq |
| .......................................................................uugaacgcagagaugAacauca................... | 6 | 1 | seq |
| .......................................................................uugaacgcagagauguGcauca................... | 3 | 1 | seq |
| .......................................................................uugaacgcagagauguacaAca................... | 2 | 1 | seq |
| .......................................................................uugaacgcagagauguacUuca................... | 2 | 1 | seq |
| .......................................................................uugaacgcagagauguacaucG................... | 5 | 1 | seq |
| .......................................................................uugUacgcagagauguacauca................... | 1 | 1 | seq |
| .......................................................................uugaGcgcagagauguacauca................... | 4 | 1 | seq |
| .......................................................................uugaacgAagagauguacauca................... | 4 | 1 | seq |
| .......................................................................uugaacgcagagGuguacauca................... | 1 | 1 | seq |
| .......................................................................uugaacgcagagauguacaucC................... | 5 | 1 | seq |
| .......................................................................Augaacgcagagauguacauca................... | 37 | 1 | seq |
| .......................................................................uugaacgcagagaCguacauca................... | 4 | 1 | seq |
| .......................................................................uuUaacgcagagauguacauca................... | 1 | 1 | seq |
| .......................................................................uugaacgcagagauCuacauca................... | 2 | 1 | seq |
| .......................................................................Nugaacgcagagauguacauca................... | 3 | 1 | seq |
| .......................................................................uugaacgcagagauguacauca................... | 10375 | 0 | seq |
| .......................................................................uuAaacgcagagauguacauca................... | 22 | 1 | seq |
| .......................................................................uugaacAcagagauguacauca................... | 2 | 1 | seq |
| .......................................................................uugaacgcagagauAuacauca................... | 1 | 1 | seq |
| .......................................................................uAgaacgcagagauguacauca................... | 10 | 1 | seq |
| .......................................................................uuCaacgcagagauguacauca................... | 2 | 1 | seq |
| .......................................................................uugaacgcagagauUuacauca................... | 1 | 1 | seq |
| .......................................................................uugaacgcagagauguacauAa................... | 3 | 1 | seq |
| .......................................................................uugGacgcagagauguacauca................... | 3 | 1 | seq |
| .......................................................................uugaacgcagagauguacaucaA.................. | 68 | 1 | seq |
| .......................................................................uugaacgcagagauguacaucac.................. | 1 | 0 | seq |
| .......................................................................uugaacgcagagauguacaucaU.................. | 40 | 1 | seq |
| .......................................................................uugaacgcGgagauguacaucac.................. | 2 | 1 | seq |
| .......................................................................uugaacgcagagauguacaucaAc................. | 2 | 1 | seq |
| ........................................................................ugaacgcGgagauguacauc.................... | 2 | 1 | seq |
| ........................................................................ugaacgcagagauguacauc.................... | 4 | 0 | seq |
| ........................................................................ugaacgcagagauguacauca................... | 36 | 0 | seq |
| .........................................................................gaacgcagagauguacauc.................... | 2 | 0 | seq |
| .........................................................................gaacgcagagauguacauca................... | 3 | 0 | seq |
